# Antibacterial therapy in treatment of newborns with perinatal sepsis

**DOI:** 10.1186/cc11799

**Published:** 2012-11-14

**Authors:** G Khanes, S Bidnenko, O Liutko

**Affiliations:** 1 Ukrainian National Paediatric Hospital OkhMatDyt, Kyiv, Ukraine; 2 Ukrainian National Orthopedic Institute, Kyiv, Ukraine

## Background

Perinatal sepsis is the leading problem for morbidity and lethality of children under 1 year old. Antibacterial therapy from the very first hours of decease development plays an important role in the treatment of perinatal sepsis. To determine the adequacy of antibacterial therapy of perinatal sepsis during 18 years we examined the etiology of perinatal sepsis in two groups of newborns with surgical pathology.

## Methods

There were 114 newborns in the first group, from 1992 to 2007. We determined the antibodies in blood to pathogenic microflora extracted from joint bursas and in 52 newborns - extracted from the abdominal cavity with peritonitis. These investigations were made using the agglutination reaction to staphylococci as well as the latex-based test for streptococci. The antibiotics were prescribed according to the results of the investigations. In the second group from 2005 to 2010, we undertook microbiological investigations of pathological material from lesion foci in 43 newborns with surgical sepsis. These investigations are devoted to the study of susceptibility and resistance of pathogenic microflora to antibiotics.

## Results

In the first group of infants, according to the data of serological investigations in the blood of newborns with bone and joint sepsis, we determined the antibodies to staphylococci in 80.3% cases, in 45.4% to staphylococci and streptococci, in 54% cases in newborns with peritonitis we determined the antibodies to *Pseudomonas aerogenes*, in 60.8% cases to *Enterobacter *spp. and in 62.5% to *Staphylococcus epidermidis*. In newborns of this group the bacterial flora were 70% and fungal in 30%. Our investigations determined the susceptibility to the limited range of antibacterial and antifungal medicines. In the second group we selected two subgroups of infants: one with sepsis as a complication of surgical deceases, and the second with bone and joint sepsis. In the first subgroup the bacterial microflora was detected in 94.7% of examined newborns and represented in 52.6% as microbial associations. In the second subgroup of newborns the bacterial flora was detected in 37.5% of cases, in the rest of the cases the flora consisted of fungi and protozoa. The analysis of susceptibility to pathogenic microflora showed that two of seven cultures of staphylococcus were methicillin-resistant *Staphylococcus aureus*, in five of eight cultures of staphylococcus they were resistant to four to 10 antibiotics, and the highest resistance to the antibiotics was detected in *P. aerogenes *(in nine of 18 cultures).

## Conclusion

Microbiological and serological diagnostics are a compulsory condition of adequate anti-inflammatory therapy for perinatal sepsis.

